# Photonic crystal band edge coupled enhanced fluorescence from magneto-plasmonic cryosoret nano-assemblies for ultra-sensitive detection

**DOI:** 10.1063/5.0251312

**Published:** 2025-04-01

**Authors:** Seemesh Bhaskar, Leyang Liu, Weinan Liu, Joseph Tibbs, Lucas D. Akin, Amanda Bacon, Brian T. Cunningham

**Affiliations:** 1 Department of Electrical and Computer Engineering, University of Illinois at Urbana-Champaign, Urbana, Illinois 61801, USA; 2 Nick Holonyak Jr. Micro and Nanotechnology Laboratory, University of Illinois at Urbana-Champaign, Urbana, Illinois 61801, USA; 3 Carl R. Woese Institute for Genomic Biology, University of Illinois at Urbana-Champaign, Urbana, Illinois 61801, USA; 4 Department of Bioengineering, University of Illinois at Urbana-Champaign, Urbana, Illinois 61801, USA; 5 Department of Chemistry, University of Illinois at Urbana-Champaign, Urbana, Illinois 61801, USA; 6 Cancer Center at Illinois, Urbana, Illinois 61801, USA

## Abstract

The fluorescence intensity associated with photon-emitters used as tags for the detection of molecular biomarkers for disease can be augmented by interfacing them with photonic crystal (PC) substrates. Although plasmonic nanomaterials are hybridized in such systems to facilitate better performance, the Ohmic losses associated with them still remain as a major bottleneck that limits the magnitude of achievable fluorescence enhancements. In this work, we present the design and synthesis of robust magneto-plasmonic, dielectric-metal, Fe_3_O_4_–Au cryosoret nano-assemblies for not only dequenching the quenched fluorescence signal but also for yielding directional steering emission output. The PC facilitates effective coupling of fluorescence emission to the PC band edge resonance and guided mode resonance by harnessing the transverse electric and transverse magnetic modes simultaneously. The resonance of the underlying PC is tailored to match the localized surface plasmon resonance of the magneto-plasmonic cryosorets (MCSs) and the emission of the radiating dipoles. The 450-fold PC band edge coupled enhancement achieved using the hottest hotspots from the MCSs in a cost-effective platform demonstrated ultra-sensitive (10 aM) sensing of a common chemical fluorophore used as a tag in biomolecular assays.

In the biological milieu, a central factor that led to the upsurge of plasmon-enhanced fluorescence (PEF) technologies is the ability of plasmonic nanoparticles (NPs) to drastically augment the local density of states (LDoS) of the radiating dipoles by concentrating electromagnetic energy into volumes smaller than the diffraction limit.^1–4^ Surface plasmon-coupled emission (SPCE) technology overcame the limitations of traditional fluorescence-based biosensors by enhancing signal collection efficiency (>50%) and minimizing background noise.^5,6^ The excited-state radiating dipoles induce or create plasmons when directly interfaced with the plasmonic substrates, in the so-called “induced plasmon effect” of PEF, and this phenomenon is well-rationalized by the radiating plasmon model.^7–9^ Although the momentum mismatch between the surface plasmon polariton (SPPs) modes and the incident plane-wave light demands the use of Kretschmann configuration,^10^ the ability of fluorophores on metallic surfaces to present wavevectors through their near-field components presented a new paradigm for effective resonant coupling captured in a series of reports by Lakowicz and co-workers.^6,11–13^ However, the far-reaching applicability of PEF and SPCE technologies are limited on account of the Ohmic losses rendered by the plasmonic substrates and also the requirement for an optical prism or objective to collect out-coupled fluorescence.^14–16^ In this context, alternative frameworks such as metal–dielectric–metal (MDM),^17^ Tamm state-coupled emission (TSCE),^18^ graphene oxide based plasmon-soliton,^19^ and guided mode resonance (GMR)-based platforms^20–22^ have been investigated. Recently, our group demonstrated the ability of photonic crystal (PC) substrates to serve as a prism-free, objective-free, and lossless all-dielectric metasurface, thereby overcoming parts of the above-mentioned limitations.

Despite the intriguing photo-plasmonic applications realized with PC substrates for fluorescence-based biosensing applications, experimental challenges yet to be resolved include (i) unavoidable Ohmic losses triggering large non-radiative channels, with concomitant quenching effects,^23–25^ (ii) chemical instability of substrates,^13^ (iii) lack of prudent engineering of nanohybrids with optimum functionalities of plasmonic NPs and lossless dielectric NPs with the latter being high refractive index (HRI) nanomaterials, to enable extreme light-confinement properties at the nanogaps between them,^26–28^ and (iv) underutilization of emitted photons from radiating dipoles that are transverse electric (TE) and transverse magnetic (TM) polarized, thereby hindering the global fluorescence enhancements.^22,29^

To address the first three challenges, this work presents a rapid, facile, linker-less, template-free synthesis of Fe_3_O_4_–Au cryosoret nano-assemblies using cryosoret nano-engineering (CSNE) technology.^30^ The HRI (RI = 2.3) Fe_3_O_4_-plasmonic Au cryosoret nano-assemblies generate multiple gap-based antennas for enhanced excitation and emission of plasmophores (plasmons + fluorophores).^2^ These materials present excellent candidates for biosensing harnessing the localized and delocalized plasmons with coherent and collective plasmon oscillations yielding regions of high field intensity in the nanogaps termed “hotspots,” demonstrating suppressed quenching.^31,32^

We begin by detailing the strategic design and application of the engineered PC substrate, leveraging the TE and TM modes supported by the PC to enhance fluorescence, thus addressing the fourth challenge. The conceptual optical schematic used in the experimentation is shown in Fig. 1(a). It can be visualized as a reverse Kretschmann configuration without an optical prism as the laser source (532 nm, 1 mW, CW laser), which directly excites the radiating dipole [Rhodamine B (RhB), molecules spin coated in PVA matrix of ~60 nm, as detailed in our earlier studies].^14,16,30^ The out-coupled emitted photons directed toward the PC far-field are collected through a 550 nm long wave pass (LWP) filter, optical fiber, and spectrometer (Ocean Optics USB2000+).

Numerical simulations by rigorous coupled-wave analysis (RCWA) were performed to evaluate the thickness conditions [Fig. 1(b)] for achieving the desired resonances of the PC. While our recent work demonstrated the utility of GMR for strong fluorescence coupling to only one of the polarizations of light, here careful analysis of the dispersion diagram was performed to tap the potential of both TM and TE modes of the underlying PC so as to minimize the loss in collection efficiency.^4,14^ From Fig. 1(c), it must be noted the PC is engineered such that its resonances (TM and TE) are far away (redshifted) from the absorbance of the dye and the laser line, ensuring minimal interference of absorption (by dye molecules) in the analysis of the fluorescence enhancements. In other words, in our experiments, the dye is directly excited using the laser and the emitted photons from the dye excites the underlying PC.^14^ In doing so, on account of the isotropic emission of the dye, resonant TM and TE modes of the PC are excited, following which the emitted photons are channeled into the far-field conforming with the dispersion properties shown Figs. 1(d) and 1(e). A scanning electron microscope (SEM) image of the PC is shown in Fig. S1 along with its 3D atomic force microscope (AFM) profile (supplementary material).

Recently, intriguing modes sustained by PCs such as Bloch surface waves (BSW),^33,34^ internal optical modes (IOM),^35^ plasmonsoliton hybrids,^19^ and GMR modes^36–38^ have been extensively investigated for the development of fluorescence based diagnostic platforms. Although the photonic bandgap (PBG) rendered by PCs presents near-zero group velocity at the band edge, their exploration has been limited to studying holographic elements in liquid crystals.^39,40^ Interfacing a gain medium at the dispersion regions supporting strong PBG edge resonances dramatically enhances the fluorescence coupling efficiency.^4^ As seen in Figs. 1(d) and 1(e), our PC is engineered so as to present counter-propagating GMR for TM and PBG for TE polarized light. Interfacing the radiating dipoles over the PC yielded the fluorescence output shown in Fig. 2(a). The polarized output is overlapped with its respective transmittance spectra with an excellent correlation between them [Figs. 2(b) and 2(c)]. A comprehensive analysis of the transmittance spectra of the PC substrates without and with PVA overcoat using different input and output polarizers is presented in the supplementary material, Figs. S2–S4. In addition, the overlap of the experimental transmittance and fluorescence for unpolarized spectra is presented in Fig. S5. While Fig. 2(b) presents coupling of fluorescence to the GMR of the PC, a very sharp emission output is seen in Fig. 2(c) as the emitted photons couple with the PBG edge of the PC. The latter coupling is substantially stronger as the LDoS is considerably boosted at the PBG edge as the photons from the radiating dipoles undergo multi-fold reflections.^40,41^ The low group velocity in this region further supports longer photon path lengths, increasing the coupling strength. The coupling occurs at the lower band edge in accordance with the high quantum yield of the radiating dipoles in this region vis-à-vis higher wavelength band edge.

Furthermore, the experimentally recorded transmittance and fluorescence spectra at different angles for TM and TE modes are presented in Figs. 2(d) and 2(e) and Figs. 2(g) and 2(h), respectively. Such an analysis clearly explains the angular pattern of the experimental fluorescence output, as the photons carry the spectral characteristics of the fluorophore and polarization selectivity of the underlying PC in accordance with the radiating GMR model.^14^ Moreover, Figs. 2(f) and 2(i) capture an excellent overlap of the experimental PCCE intensity (shaded background color) with the simulated dispersion diagram (dotted blue stars). As a 550 nm LWP filter was used to collect the out-coupled emission, the spectral signal closer to this wavelength presents irregular distribution of coupling efficiency (seen in wavelength regions 550–600 nm of the dispersion diagram). Additional analysis of the out-coupled fluorescence spectra and the transmittance spectra is presented in the supplementary material, Fig. S6. While these measurements yielded ~50-fold enhancement in the fluorescence (compared to that over the glass substrate under identical conditions), metallo-dielectric nanohybrids synthesized using CSNE were utilized to further augment the signal intensity, as discussed in the following.

Among several plasmonic nano-systems explored in the broad domain of point-of-care diagnostics, plasmonic AuNPs and their associated nanohybrids have established a strong foothold on account of high chemical stability, ease of biofunctionalization, and high electron density (≈5.90 × 10^16^ m^−3^).^13,24^ Although the fluorescence spectroscopic and microscopic modalities are widely explored for biosensing applications using the AuNPs, the limitations pertaining to quenching in the so-called “zone of inactivity” (<5 nm) and associated experimental artifacts has remained a longstanding challenge.^24,32^ Different approaches including core–shell architectures, sharp-edged nano-systems, conical topographies with nano-holes, to name a few have been employed to overcome such drawbacks.^13,42^ In the recent past, cryosoret nano-assemblies have emerged as exceptional substrates in comprehending the photo-plasmonic coupling and related physicochemical interactions at nanoregimes due to generation of “hottest hotspots.”^15,30,42^ Further more, metal–dielectric nanohybrids outperform the metal–metal and dielectric–dielectric counterparts in augmenting the EM field intensity, especially when the dielectric is composed of HRI nanomaterial on account of the generation of less lossy plasmonic hotspots.^28,42–44^ Moreover, the magneto-plasmonic nano-assemblies sustain electric and magnetic hotspots.^45–48^ In the past, although metal–dielectric interfaces including dielectrics such as SiO_2_, TiO_2_ (nanorods and nanospheres), and graphene analog sustaining π-plasmons have demonstrated excellent sensing capabilities,^30^ cryosoret nano-assemblies comprising a magnetic material with HRI is not reported hitherto.^49^

In light of these observations, in this work, we present the synthesis of HRI Fe_3_O_4_– plasmonic Au cryosoret nano-assemblies, as shown in Fig. 3(a), using a well-established adiabatic cooling methodology.^30,50^ In brief, the colloidal aqueous solutions of AuNPs (~20 nm) and Fe_3_O_4_ NPs (~20 nm) were mixed in a 20 ml glass vial (volume = 10 ml, diameter = 2.5 cm) in 1:1 ratio and immersed in LN2 for predefined time intervals. Following this exposure, the samples were thawed to room temperature, centrifuged, washed, and used for further analysis. The number of NPs per magneto-plasmonic CSs (MCSs) is tunable by changing the adiabatic cooling time, where 15 s, 30 s, 1, 2, and 3 min of −196 °C LN2 exposure yields 2 (±1), 5 (±2), 7 (±2), 9 (±3), and 13 (±3) number of NPs per assembly. While the representative transmission electron microscopy (TEM) and high-resolution TEM (HRTEM) images are shown in Figs. 3(c)–3(g) and 3(h)–3(l), respectively, multiple TEM images for each of the sample variants are presented in the supplementary material (Fig. S7). The HRTEM image indicates d-spacing (characteristic lattice fringes) of metallic Au (blue) and dielectric Fe_3_O_4_ (red) in accordance with the respective reference codes from the Inorganic Crystal Structure Database (ICSD) (Au: 98-061-1625, and Fe_3_O_4_: 98-008-5807).

The localized surface plasmon resonance (LSPR) spectra of all the MCSs (1–5) are presented in Fig. 3(b), shown alongside the transmittance dip corresponding to the unpolarized mode of the PC. The LSPR of the plasmonic AuNPs shifts from ~525 to 630 nm as the MCSs are formed. This redshift is a direct result of the MCS morphology presenting high density of three-dimensionally^51^ distributed magneto-plasmonic hotspots (as seen in TEM images). In order to understand the signal contrast presented by the MCS variants, photonic resonator interferometric scattering microscopy (PRISM) imaging was carried out as detailed in our earlier studies.^52,53^ The representative processed images of different MCSs obtained with PRISM are shown in Figs. 3(m)–3(q) and the calculated signal contrast is shown in Fig. 3(r). From these PRISM measurements, we observe that the contrast signal that characterizes the scattering efficiency of the nanomaterial increases from MCS1 to MCS4, and then decreases, indicating that the MCS4 presents the optimum number of NPs in the cryosorets to present the highest scattering. When these MCSs are integrated with the PC, which is coupled with radiating dipoles, interesting plasmon–photon coupling phenomena occur. In this system, the excited fluorophores primarily induce plasmons in the MCSs (the induced plasmon effect of PEF).^2,6^ Another key mechanism contributing to plasmon excitation arises from the GMR of the PC, which strongly resonates with the LSPR of the MCSs. The intriguing results obtained from the MCS-PC interface are shown in Fig. 4, highlighting these coupling effects.

The photonic crystal coupled emission (PCCE) peaks seen in Fig. 4(b) are classified as photonic-crystal band-edge coupled emission (PBCE) and photonic-crystal GMR coupled emission (PGCE) in accordance with their coupling to the band edge and GMR, respectively. The fluorescence enhancements calculated as the direct ratio of the fluorescence intensity counts obtained using the PC to that of a glass interface is shown in Fig. 4(a) along with the percentage polarizations in Fig. 4(c). The modulation in the fluorescence signal intensity achieved using different MCSs (1–5) is shown in Fig. 4(b). While the fluorescence signal is quenched for the use of pristine AuNPs (compared to blank),^24,32^ the intensity increases with the use of MCSs. The upsurge in fluorescence reaches the maximum for MCS4 and further decreases for MCS5, indicating that the variant MC4 presents an optimum balance of the number of NPs per assembly (excessive AuNPs per assembly as in MCS5 would show predominant quenching effects).^30^ It is worth mentioning that the experimentally observed trend in the fluorescence enhancements for the MCS(1–5) [Fig. 4(a)] are in excellent agreement with the PRISM-detected signal contrast (signifying the scattering efficiency) trend observed in Fig. 3(r), hence cross-validating our experimental fluorescence enhancements. Figure 4(d) shows the spectra for unpolarized, TM-, and TE-polarized emissions obtained by placing the polarizer between the detector and the PC, for the sample that yielded the highest fluorescence enhancements (the corresponding overlap plots with the experimental transmittance is captured in Fig. S8). In addition, the overlap between the experimentally observed fluorescence spectra and the simulated transmittance data [shown in Figs. 4(e) and 4(f)] further confirms the nature of the out-coupled emission, where the photons carry the spectral signature of the radiating dipoles and the polarization selectivity of the PC.

From Fig. 4(a), it is seen that MCS4 yielded >450-fold increase in the fluorescence intensity for PBCE compared to that obtained over the glass substrate (free space spectra shown in Fig. S9), under identical conditions. The data pertaining to fluorescence enhancements and percentage polarizations obtained using AuCSs and MCSs for all the variants are captured in Figs. S10 and S11. The high fluorescence output is attributed to the large optical cross sections of the magneto-plasmonic cryosorets that exceeds that of the bare fluorophores by 4–5 orders of magnitude.^2,54^ The emission from the proximal radiating dipoles is hence dramatically increased as the radiative decay increases and lifetime decreases.^55,56^ This variant MCS4 that yielded the highest fluorescence enhancement was utilized for the detection of the typical SPCE and PCCE reporter molecule, RhB, by introducing a linear reduction in the concentration of the dye. In the hybrid nanocavity interface using MCS4, different concentrations of RhB was studied, ranging from 1 mM to 0.01 fM [Fig. 4(g)].

Expermentally, it was observed that for concentrations higher than 1 μM, the fluorescence enhancements remained ~400 to 450 (not clearly distinguishable) and drastically decreased for concentrations of less than 1 μM. This is on account of the ability of the MCSs to overcome the quenching effects for concentrations of more than 1 μM. Furthermore, it is observed that distinctly two linear ranges may be differentiated and extracted from the experimental data, with the sensitivity presented as the slope being 54 ± 2.19/dec and 15 ± 0.48/dec for range 1 [1 μM to 0.01 nM, Fig. 4(h)] and range 2 [0.01 nM to 0.01 fM, Fig. 4(i)], respectively, with high reproducibility (standard deviation presented for triplicate measurements). Importantly, it is worth mentioning that the attomolar (10 aM) concentration of the RhB corresponds to concentrations very close to the single-molecule limit of detection.^35^ With these proof-of-concept observations, the PC substrate is envisaged to support a better limit of detection completely preventing the quenching effects, and such explorations are under way.

The sequence of optical events occurring at the radiating dipole–MCS–PC hybrid interface can be summarized as follows: (i) the laser excites the radiating dipoles, which then emit photons; (ii) these emitted photons excite the GMR of the underlying PC according to the radiating GMR model;^14^ (iii) simultaneously, the emitted photons also excite the plasmons in the MCSs, initiating the induced plasmon effect of PEF;^2,6^ and (iv) by carefully engineering the resonance conditions of the MCSs and the GMR, a new mechanism emerges where the excited GMR further excites the plasmons in the MCSs, as they are coupled with the PC [Fig. 3(b)]. This interplay results in the enhancement of nanogap-induced antenna effects, which outperforms the detrimental surface-induced quenching effects.^31^ In particular, the close proximity of magnetic and plasmonic NPs within the assembly generates hybridized higher-order modes that lead to extremely high field confinement within the nanogaps of the MCSs.^31,57^ Moreover, the infinitesimal nanogaps formed by interfacing the MCSs over the PC give rise to multiplicative effects due to the resonant coupling of multiple hotspots in the MCSs with the GMR of the PC.

Bragg mirror-based PC interfaces, previously explored for high-sensitivity detection via Bloch surface waves and internal optical modes,^15,16,35^ require optical prisms or objectives for realizing the phase matching condition. To overcome this, we developed a prism-free technology using photonic crystal (PC) grating interface, where the diffraction properties of the underlying PC is exploited to observe PC resonances starting from zero degree in the dispersion diagram (wavelength vs angle).^14^ In this work, our modified PC geometry enables simultaneous tailoring of TE and TM resonances for enhanced fluorescence emission coupling with the band edge modes and the GMR, respectively. The fluorescence measurements are largely affected by the local density of optical states (LDOS) available in the close proximity of the fluorophores.^58^ Moreover, the LDOS is directly proportional to the quality factor (QF) of the system under consideration, hence making the QF an important metric, based on which several photonic substrates are engineered.^59,60^ QF is defined as the ratio of the wave length maximum of the resonant peak to that of the full width half maximum (FWHM) of the resonant peak.^36,61^ In the case of the photonic-crystal band-edge coupled emission (PBCE) observed in this work, the experimentally calculated QF is ~200. This indicates a high-quality factor resonant PC interface with a high LDOS, hence explaining the high fluorescence enhancements observed experimentally.

Conventionally explored magneto-plasmonic interfaces report the magnetic NPs as part of the core in core–shell morphologies, thereby concealing its full magnetic flux potential to the surrounding micro-nano-environment. Nano-assembly geometry circumvents such inadequacies as the surfaces of both the plasmonic and magnetic nanomaterials are exposed and readily accessible to build desired biomolecular interactions. Moreover, the ability of magneto-plasmonic cryosorets to couple with the PBG edge as well the GMR of the PC harnessing both the TE and TM modes demonstrated here presents newer insights into the fluorophore-PC interactions valuable for technologies associated with biosensor development (Fig. S12). We believe that the unique fluorophore–cryosoret nanoassemblage presented here paves the way for the development of frugal and highly desirable strategies for the quantification of environmentally and physiologically relevant analytes at trace concentrations for use in point-of-care diagnostics.

The supplementary material provides comprehensive characterization of the substrates and nano-systems under investigation using AFM, TEM, fluorescence, and transmittance measurements. The free space (FS), PCCE intensity analysis, and the transmittance data analysis under different input and output polarizations (TM and TE) of light are discussed for different sample variants, including bare AuCSs and magento-plasmonic cryosorets (MCSs). The scope and perspectives of the current research is outlined presenting opportunities for future work in this direction.

The authors acknowledge funding from the National Institutes of Health (Grant Nos. R01AI159454, R01CA227699, R01AI139401, R01EB029805, and RadXRad Program grant U01AA029348) and the National Science Foundation (Grant Nos. NSF RAPID 20–27778, CBET 19–00277, and CBET 22–32681). Financial support was also provided by the Cancer Center at Illinois. J.T. was supported by the Illinois Distinguished Fellowship and the National Science Foundation graduate research fellowship. S.B. was supported by a postdoctoral fellowship from the Woese Institute for Genomic Biology. S.B. thanks the research scientists, Kathy Walsh, Ying He, Jade Wang, Duncan Nall, Umnia Doha, and Glenn Fried for their support and research inputs in characterization of materials. The authors acknowledge the support from the following instruments: *Asylum Research MFP-*3*D AFM*; *Hitachi S-*4800 *High Resolution SEM*; *Au-Pd Sputter Coater - Emscope SC* 500*; JEOL* 2100 *CRYO TEM*, the associated staff and research scientists at Materials Research Laboratory, the Grainger College of Engineering, UIUC. The authors thank the feedback provided by all the members of the *Nanosensors* group, HMNTL, during scientific discussions.

## Supplementary Material

Supplementary Information

## Figures and Tables

**FIG. 1.**
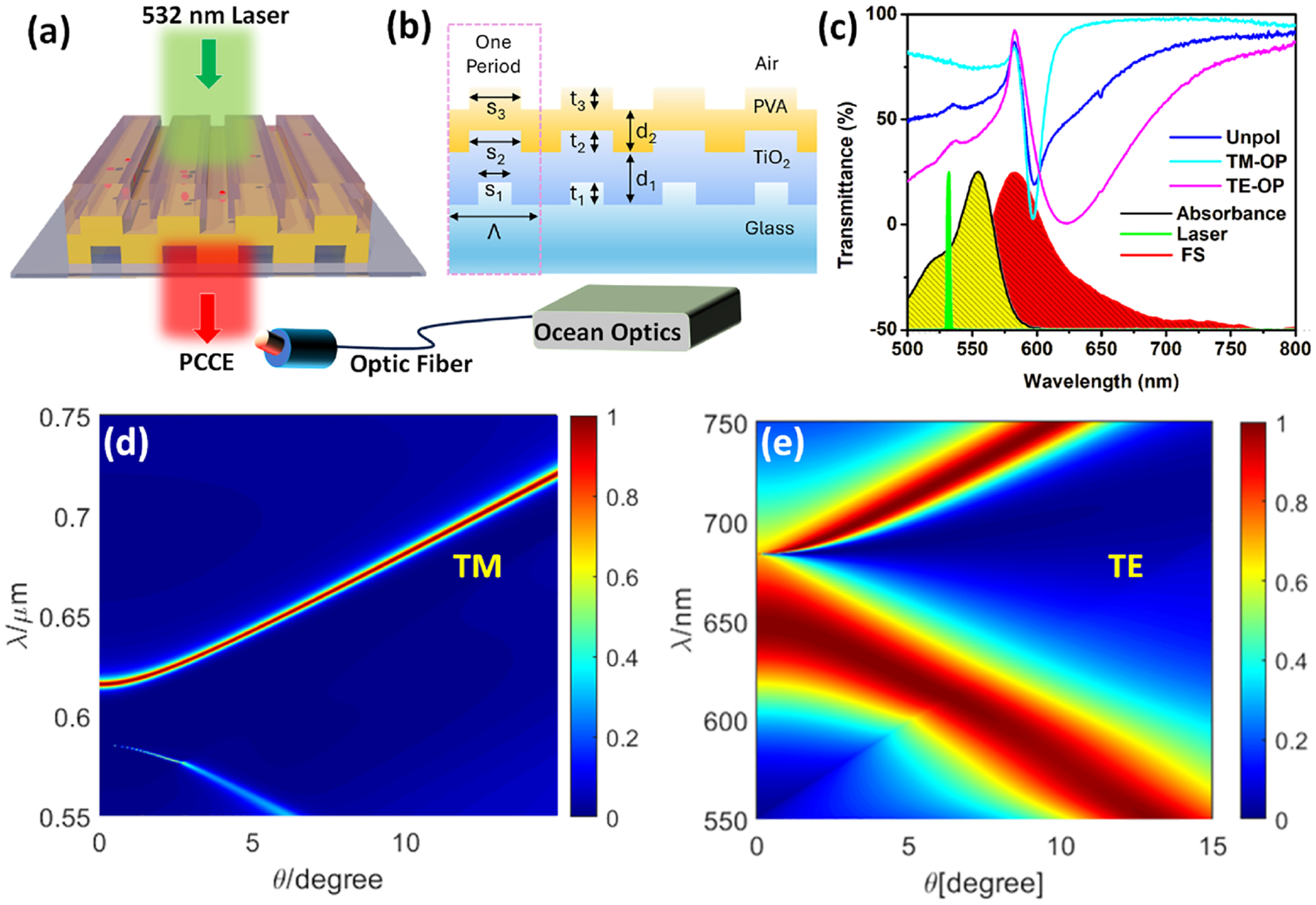
Optical configuration, PC design, dispersion diagrams, optical, electron microscopy, and topography characterization. (a) Representative optical framework presenting the excitation and emission collection configuration. (b) Conceptual schematic of the PC surface, with the parameters being Λ = 380 nm, S_1_ = 190 nm, S_2_ = S_3_ = 247 nm; t_1_ = t_2_ = t_3_ = 97 nm, d_1_ = 98.5 nm, and d_2_ = 60 nm. (c) Transmittance resonance dips of the PC overlapped with the laser line, absorbance, and emission of RhB, all collected experimentally (OP: output, where the polarizer is placed between the PC and the detector, essentially in the output region of the far-field emission). Wavelength vs angle, dispersion diagrams for (d) transverse magnetic (TM), and (e) transverse electric (TE) modes sustained by the PC obtained by RCWA.

**FIG. 2.**
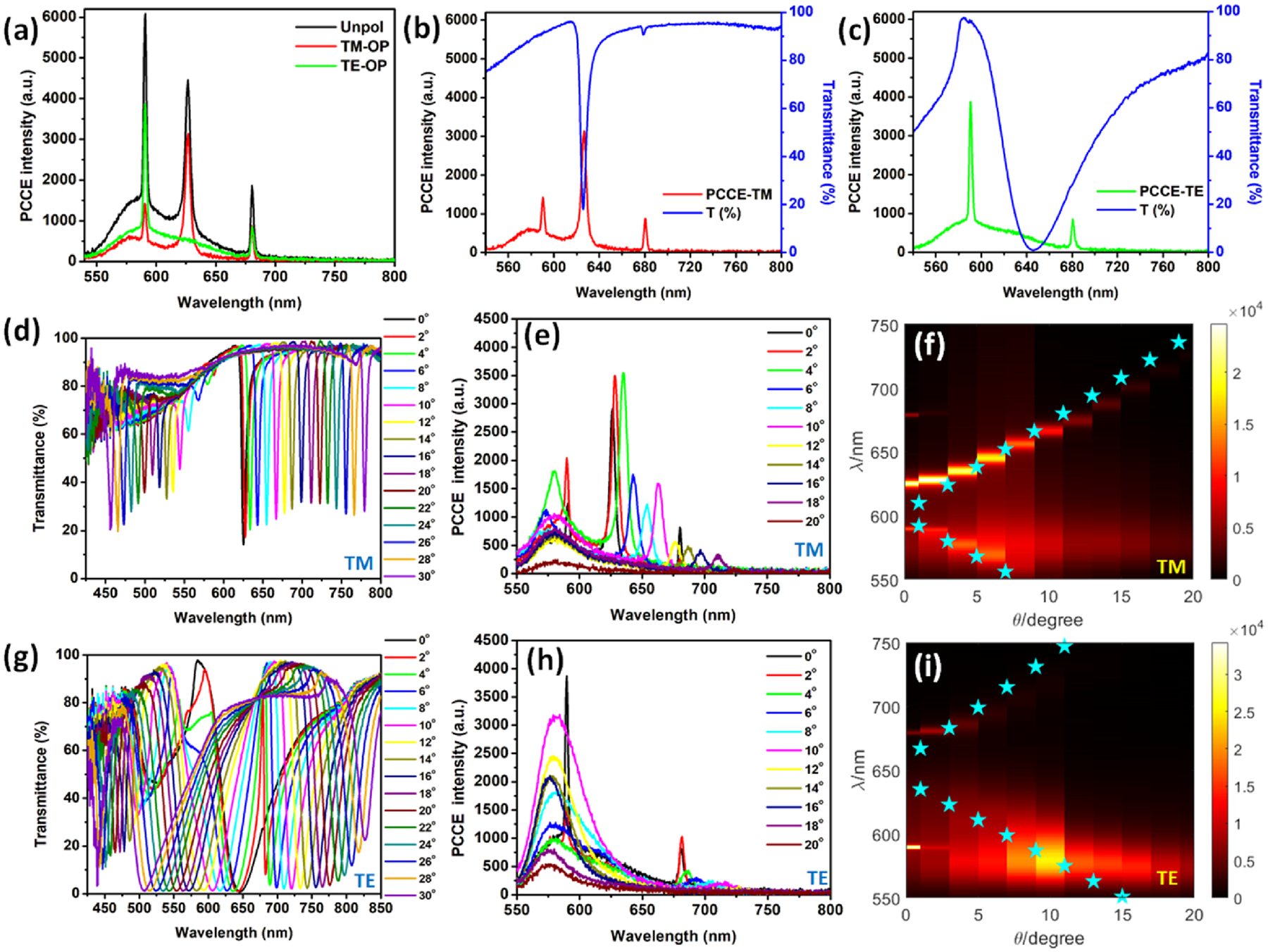
Analysis of experimental and theoretical transmittance data with the experimental fluorescence data. (a) PCCE intensity spectra shown with TE and TM coupled PCCE. Overlap of the experimentally obtained transmittance spectra and the experimentally obtained fluorescence spectra for (b) TM and (c) TE polarized out-coupled spectra. Experimental transmittance and fluorescence recorded at different angles for TM and TE modes are presented in panels (d) and (e) and (g) and (h), respectively. Overlap of the experimental PCCE intensity with the simulated dispersion diagram where the simulated plot in Figs. 1(d) and 1(e) is represented using the dotted blue stars and the fluorescence spectra is represented using the shaded background color for (f) TM and (i) TE modes, respectively.

**FIG. 3.**
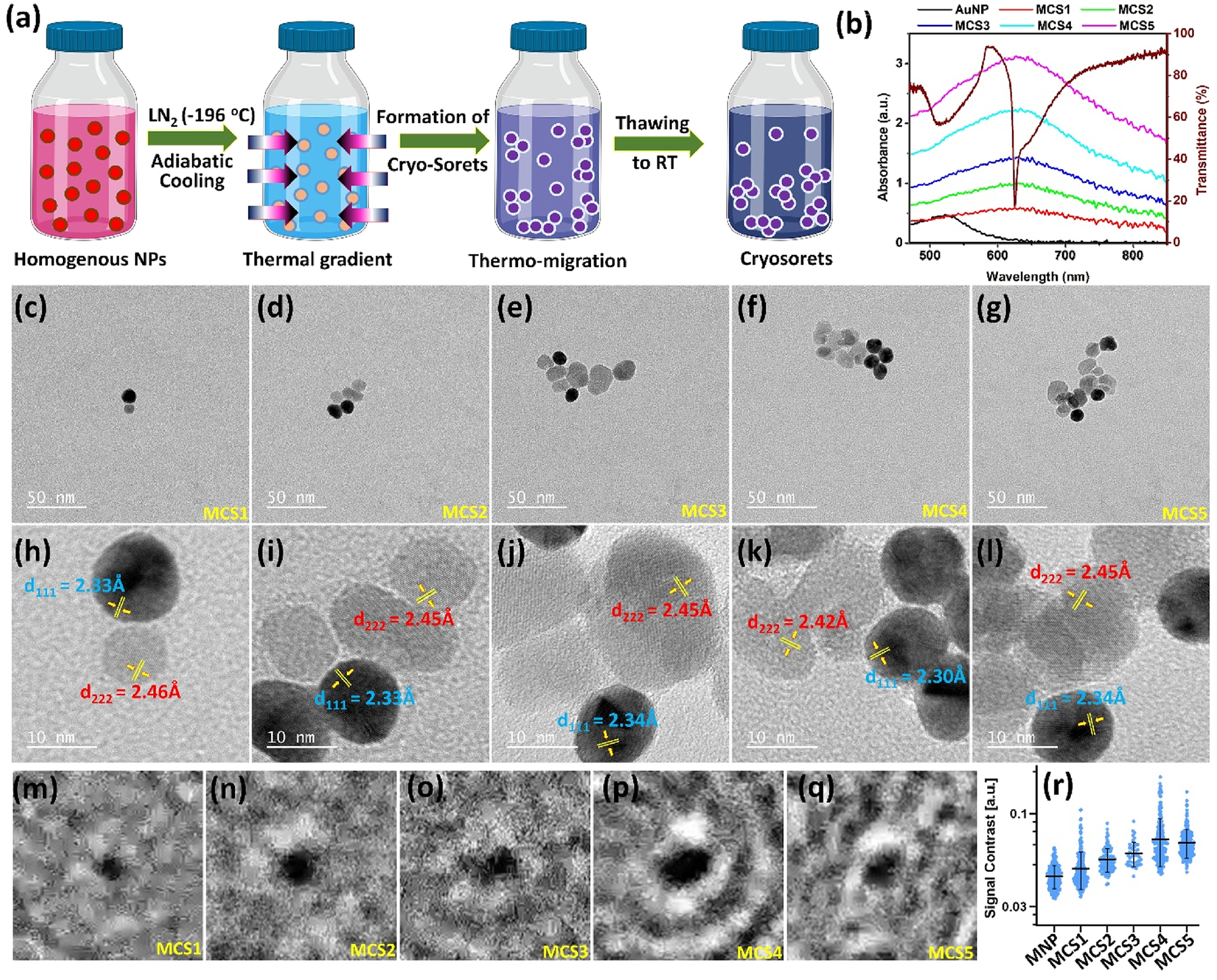
Synthesis, optical, and electron microscopy characterization of magneto-plasmonic cryosorets (MCSs). (a) Schematic depicting the formation of cryosorets under adiabatic cooling at −196 °C, where thermomigration of NPs occurs, followed by thawing of the nano-assemblies to room temperature. (b) Redshift in the LSPR of the pristine AuNPs observed with increasing the number of NPs per MCSs assembly, with excellent overlap with the unpolarized mode of the PC. (c)–(g) TEM images and respective (h)–(l) HRTEM images of the MCSs obtained by subjecting the NPs to 15 s, 30 s, 1, 2, and 3 min of −196 °C LN2 environment. (m)–(q) Selected processed images of different MCS variants obtained with PRISM. (r) The calculated signal contrast of the MNP and MCS variants.

**FIG. 4.**
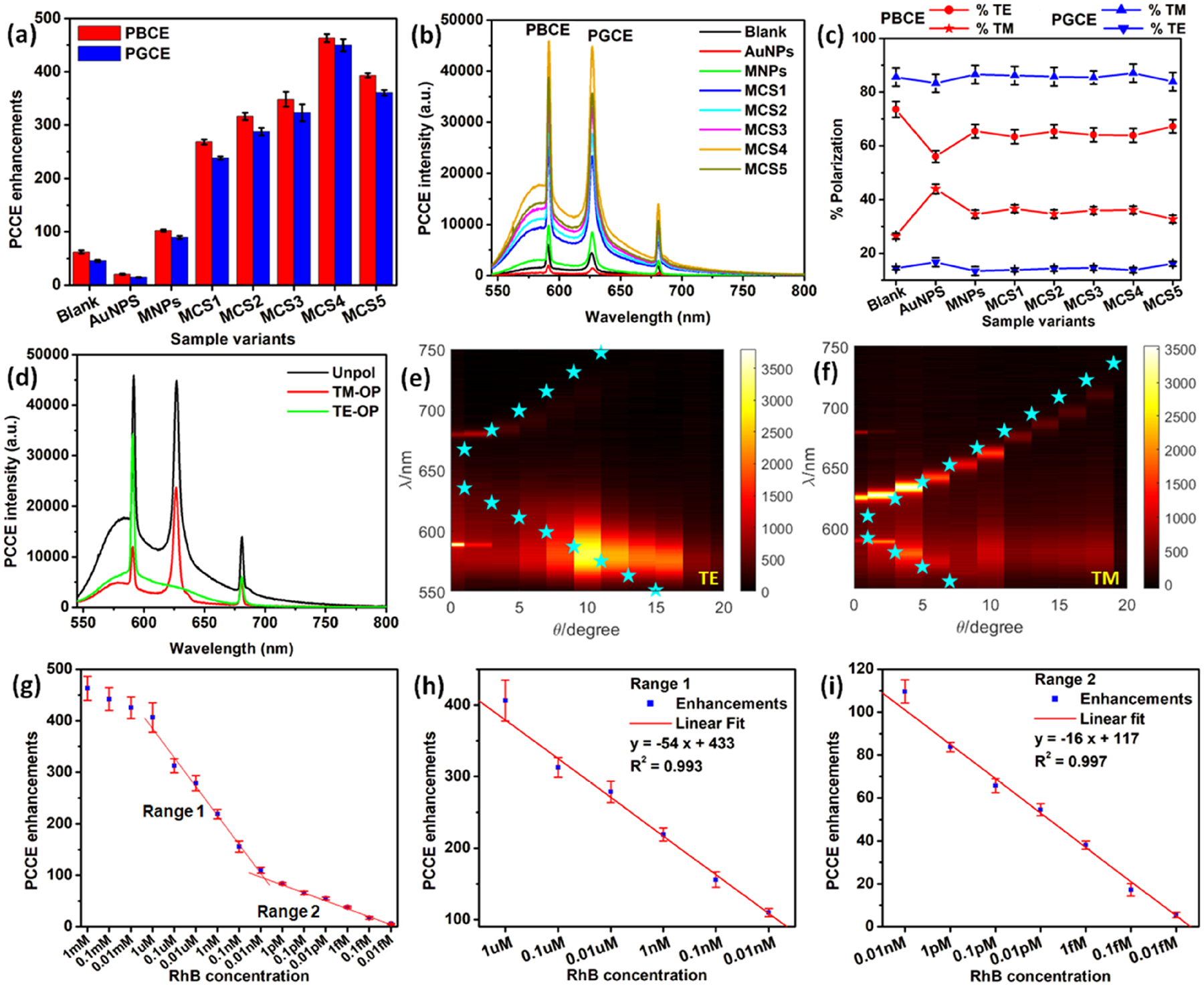
PCCE enhancements, dispersion diagrams, and dose-dependent RhB detection. (a) Photonic-crystal band-edge coupled emission (PBCE) and photonic-crystal GMR coupled emission enhancements. (b) Quenching by AuNPs and dequenching by MCSs observed experimentally for different MCSs (1–5) with the respective percentage polarizations given in panel (c). (d) PCCE emission spectra for unpolarized, TE, and TM polarized emission recorded for the sample yielding highest fluorescence enhancements, MCS4. Overlap of the experimental PCCE intensity with the simulated dispersion diagram where simulated data are represented using the dotted blue stars and the fluorescence spectra are represented using the shaded background color for (e) TE and (f) TM modes, respectively. Linear calibration plots for sensing RhB in the ranges from (g) 1 mM to 0.01 fM (full range), (h) 1 μM to 0.1 nM, and (i) 0.01 nM to 0.01 fM.

## Data Availability

The data that support the findings of this study are available from the corresponding author upon reasonable request.
