# Supplementary material for: Photonic crystal band edge coupled enhanced fluorescence from magneto-plasmonic cryosoret nano-assemblies for ultra-sensitive detection
Source: APL Mater. Author manuscript; Available in PMC 2026 May 22. (PMC13193251; doi:10.1063/5.0251312)
Supplement: Supplementary Information [file NIHMS2175798-supplement-Supplementary_Information.docx]

**Supplementary Information**

**Photonic Crystal Band Edge Coupled Enhanced Fluorescence from Magneto-Plasmonic Cryosoret Nano-assemblies for Ultrasensitive Detection**

Seemesh Bhaskar,^1,2,4^ Leyang Liu,^1,2#^ Weinan Liu,^1,2#^ Joseph Tibbs,^2,3^ Lucas A Akin,^2,5^ Amanda Bacon,^2,3^ and Brian T. Cunningham^1,2,3,4,5,6*^

^1^Department of Electrical and Computer Engineering, University of Illinois at Urbana-Champaign, Urbana, IL 61801, USA

^2^Nick Holonyak Jr. Micro and Nanotechnology Laboratory, University of Illinois at Urbana-Champaign, Urbana, IL 61801, USA

^3^Department of Bioengineering, University of Illinois at Urbana-Champaign, Urbana, IL 61801, USA

^4^Carl R. Woese Institute for Genomic Biology, University of Illinois at Urbana-Champaign, Urbana, IL 61801, USA

^5^Department of Chemistry, University of Illinois at Urbana-Champaign, Urbana, IL 61801, USA

^6^Cancer Center at Illinois, Urbana, IL 61801, USA

*Corresponding author: Brian T. Cunningham; Email: [bcunning@illinois.edu](mailto:bcunning@illinois.edu)

^#^ Leyang Liu and Weinan Liu contributed equally to this work.


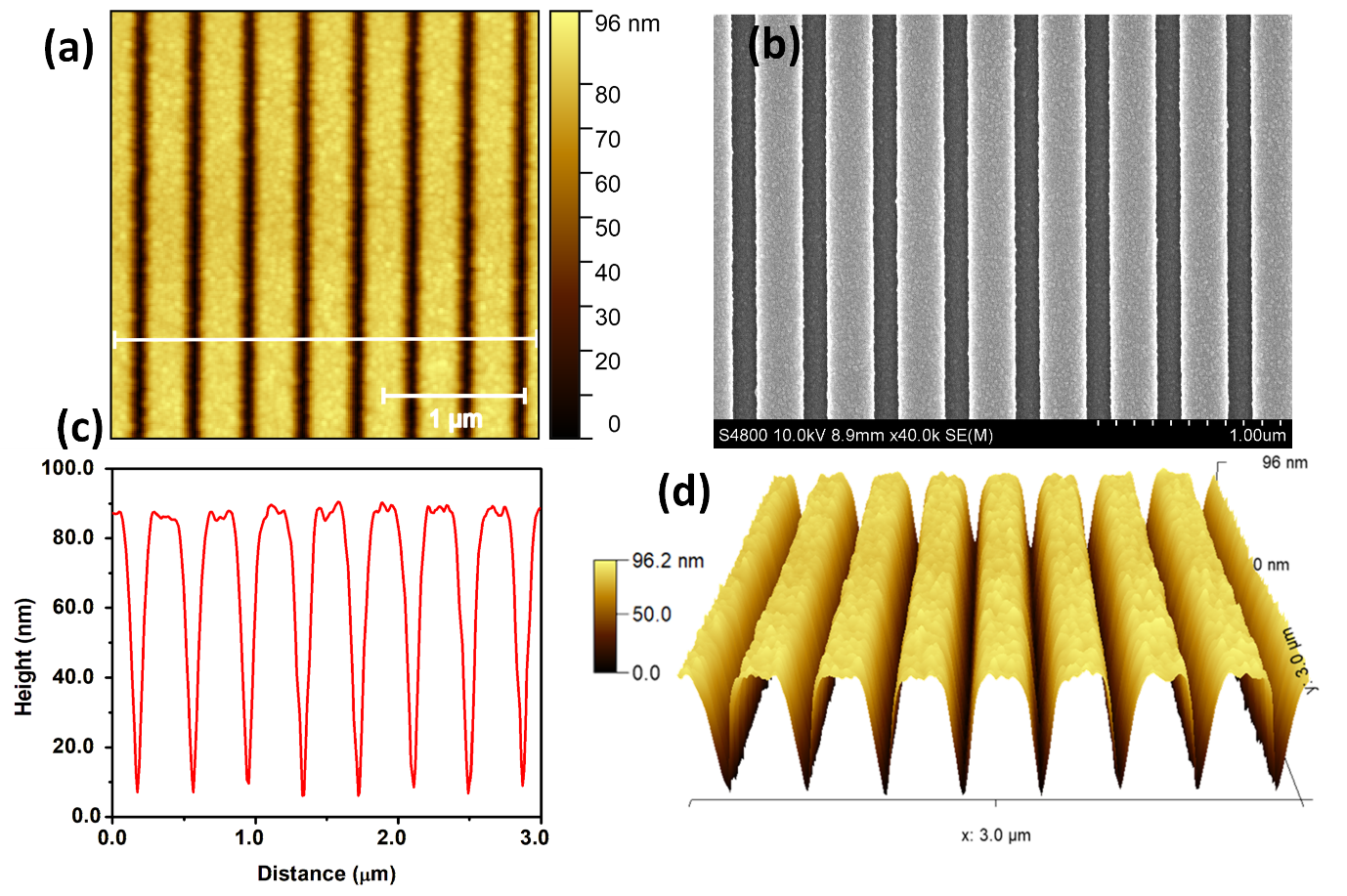


**Figure S1.** **AFM and SEM analysis of PC**: (a) AFM Surface top view, (b) SEM surface top view, (c) height profile along the white line drawn in Figure S1a. (c) 3D profile view of the PC shown in Figure S1a.


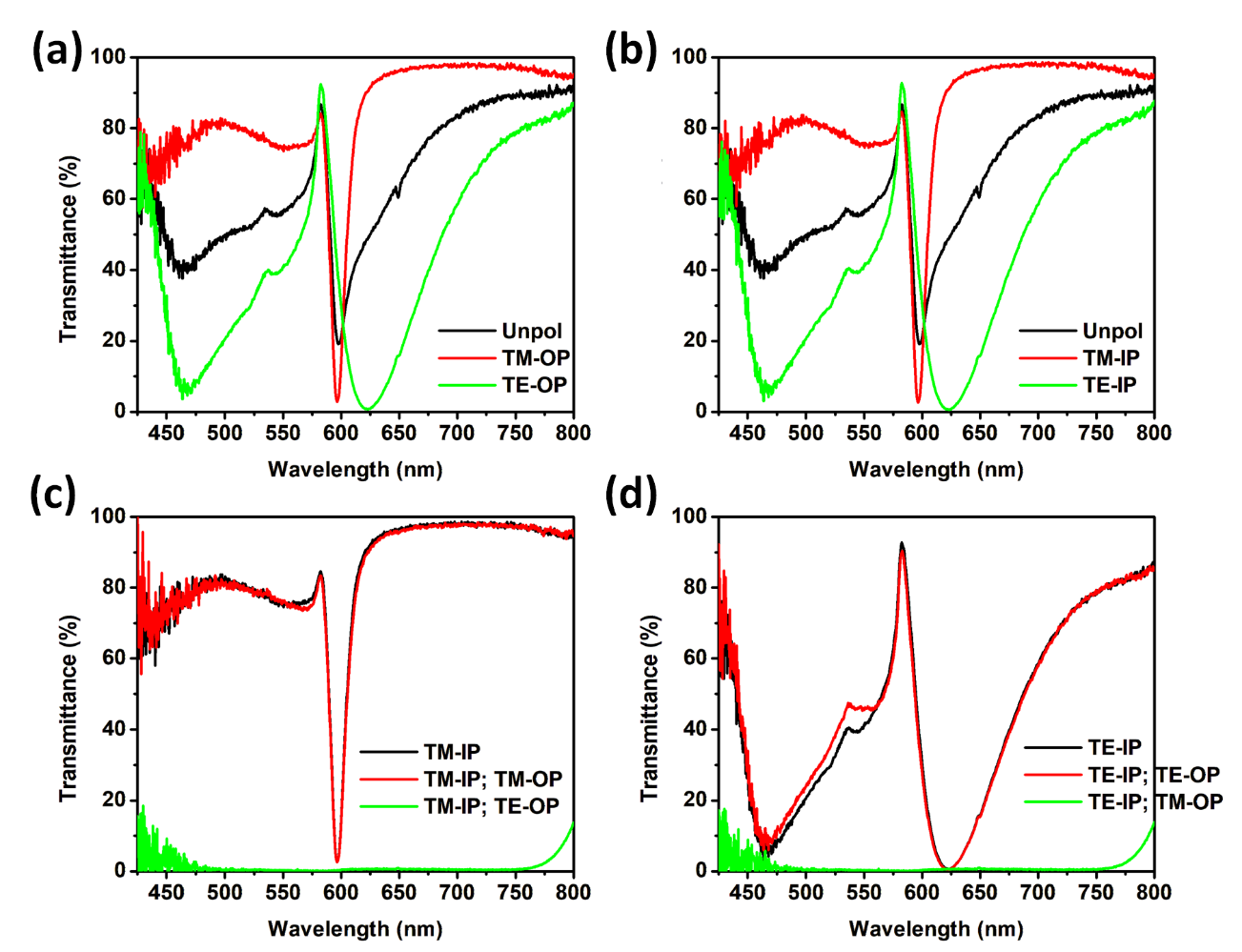


**Figure S2. Transmittance spectra for PC at different input and output polarizations.** Transmittance vs wavelength spectra for (a) unpolarized, TE and TM output (OP), (b) unpolarized, TE and TM input (IP), (c) TE and TM output for a fixed input (IP) being TM, (d) TM and TE output for a fixed input being TE. These are for samples without the PVA overcoat.

IP, which refers to input means that the polarizer is being placed between the light source and the PC. Similarly, OP, which refers to output means that the polarizer is being placed between the PC and the detector. Figure S2a and S2b has a very similar profile, indicating that the polarizers are performing optimally and is not introducing any disproportionate errors in the spectra (whether they are inserted before the light hits the PC or after the light passes through the PC). While the IP polarizer showcases if there is any effect of the polarizer itself, the OP polarizer presents the polarization selectivity of the underlying PC as the light is now being passes through the PC. Further, it is important to note that while using a similar polarizer in both input and output regions yields a resonance mode of the PC, using opposite polarizers blocks all the resonances, as expected. This trend is similar to the PC substrates coated with the PVA matrix as well, shown in Figure S3.


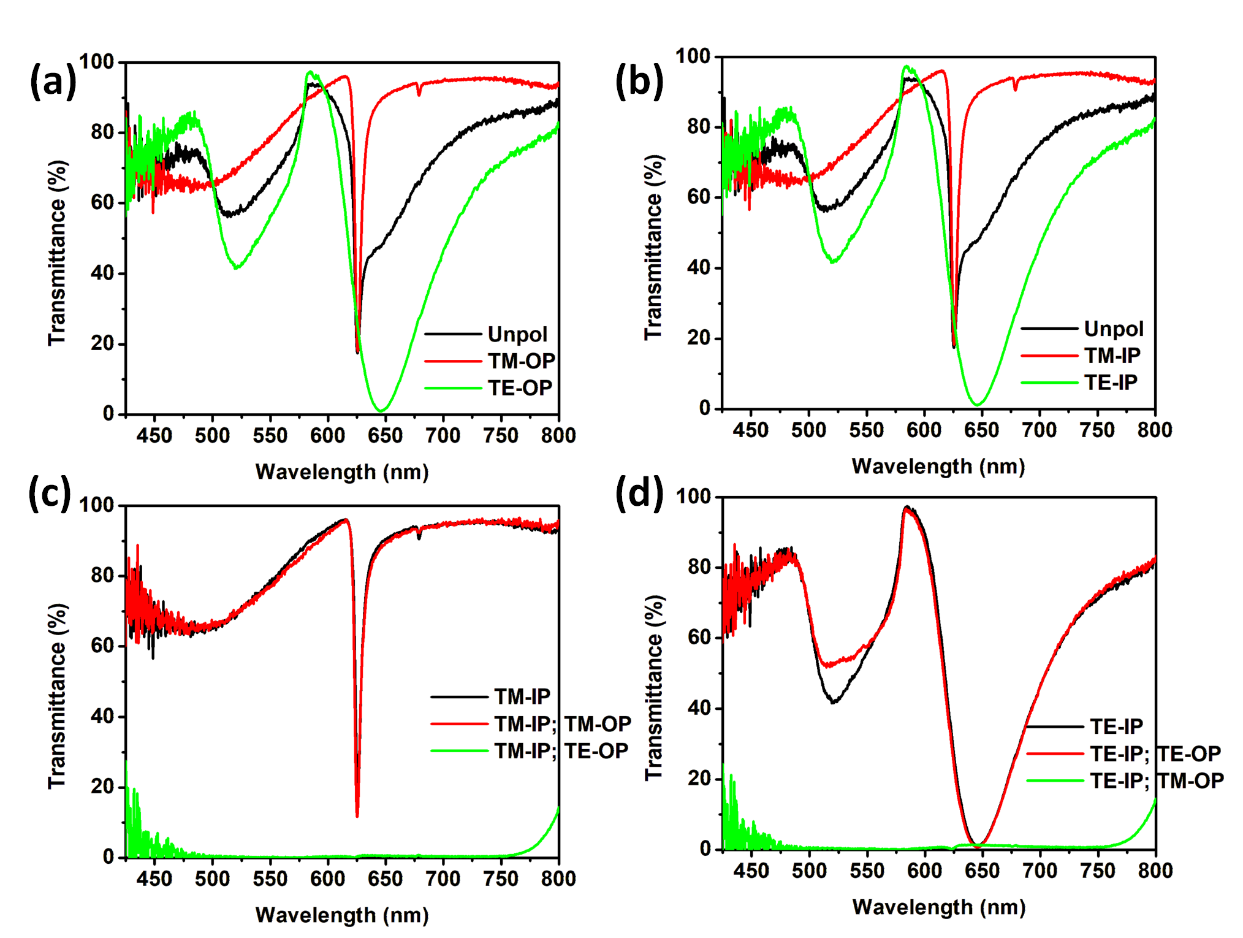


**Figure S3. Transmittance spectra for PC at different input and output polarizations.** Transmittance vs wavelength spectra for (a) unpolarized, TE and TM output (OP), (b) unpolarized, TE and TM input (IP), (c) TE and TM output for a fixed input (IP) being TM, (d) TM and TE output for a fixed input being TE. These are for samples with the PVA overcoat on the PC.


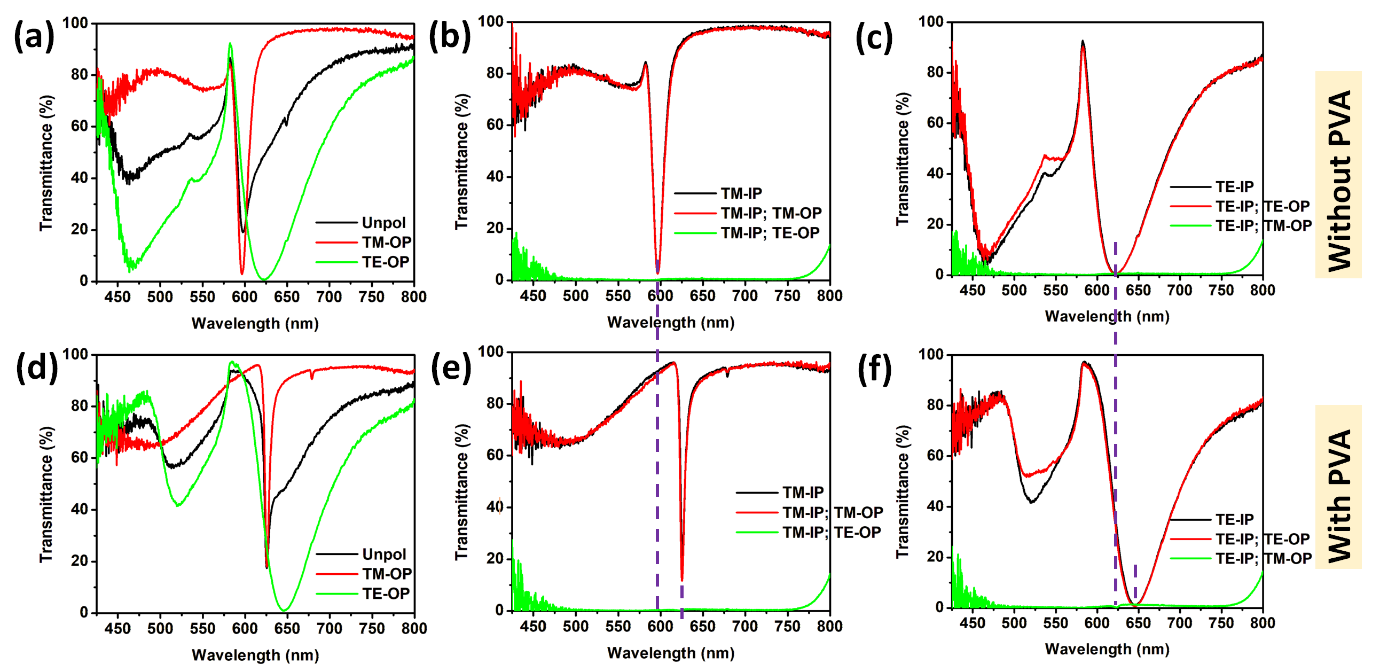


**Figure S4. Transmittance spectra for PC at different input and output polarizations.** For PC samples without PVA, transmittance vs wavelength spectra for (a) TE and TM output (OP), (b) TE and TM output for a fixed input (IP) being TM, (c) TM and TE output for a fixed input being TE. For PC samples with PVA, transmittance vs wavelength spectra for (d) TE and TM output, (e) TE and TM output for a fixed input being TE, (f) TM and TE output for a fixed input being TM. A slight red-shift in the resonances of the PC is observed on account of additional thickness rendered by PVA overcoat.


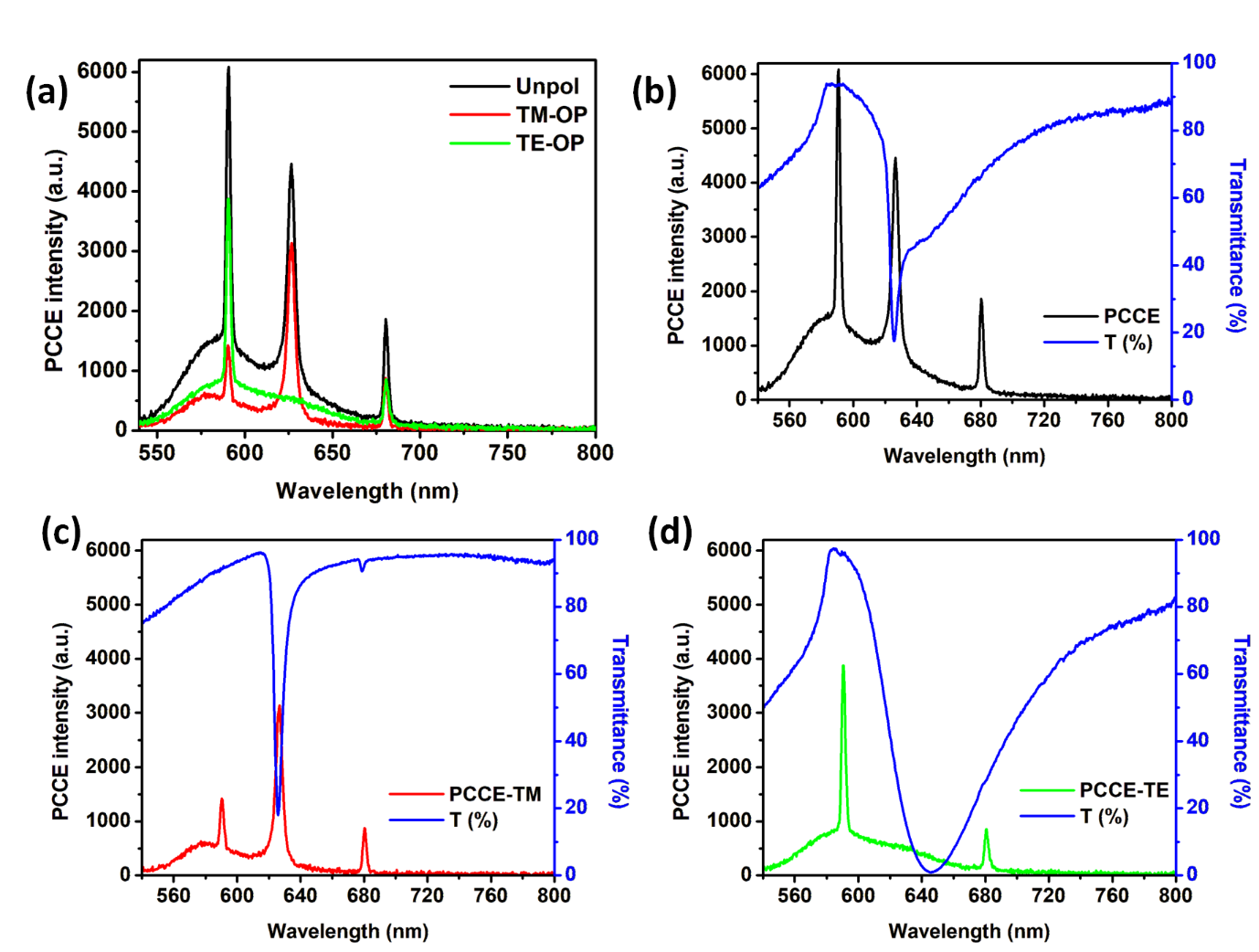


**Figure S5. Complete analysis of experimental transmittance data with the experimental fluorescence data**. (a) PCCE intensity spectra shown with TE and TM coupled PCCE. Overlap of the experimentally obtained transmittance spectra and the experimentally obtained fluorescence spectra for (b) unpolarized, (b) TM, (c) TE polarized out-coupled spectra. [please note that the Figures S5a, 5c, 5d are part of figure 2 in the manuscript and is presented here again along with Figure S5b to present better clarity of the coupling between PC and radiating dipoles].

Figure S6 presents interesting inferences which are worth highlighting. Figures S6 presents the PCCE intensity and the modulations observed in the same by introducing TM and TE polarizers at the emission end (between the PC substrate and the detector). Inserting the polarizers results in out-coupling of modes that are allowed by the PC in accordance with the modes shown in the dispersion diagram (see figures 1e and 1f of the manuscript). In order to re-emphasize this aspect, the transmittance of the PC without the dye is shown in Figure S6d. There is an excellent agreement between the results presented by PCCE spectral information in Figure S6a and the corresponding transmittance data for the same PC shown in Figure S6d. In order to facilitate effective visual comparison, the Figures S6d is arranged vertically below the Figures S6a. Also, the coupling of the TE mode to the PBG edge is evident as seen by green colored emission spectra (Figure S6a) and transmittance spectra (Figure S6d).


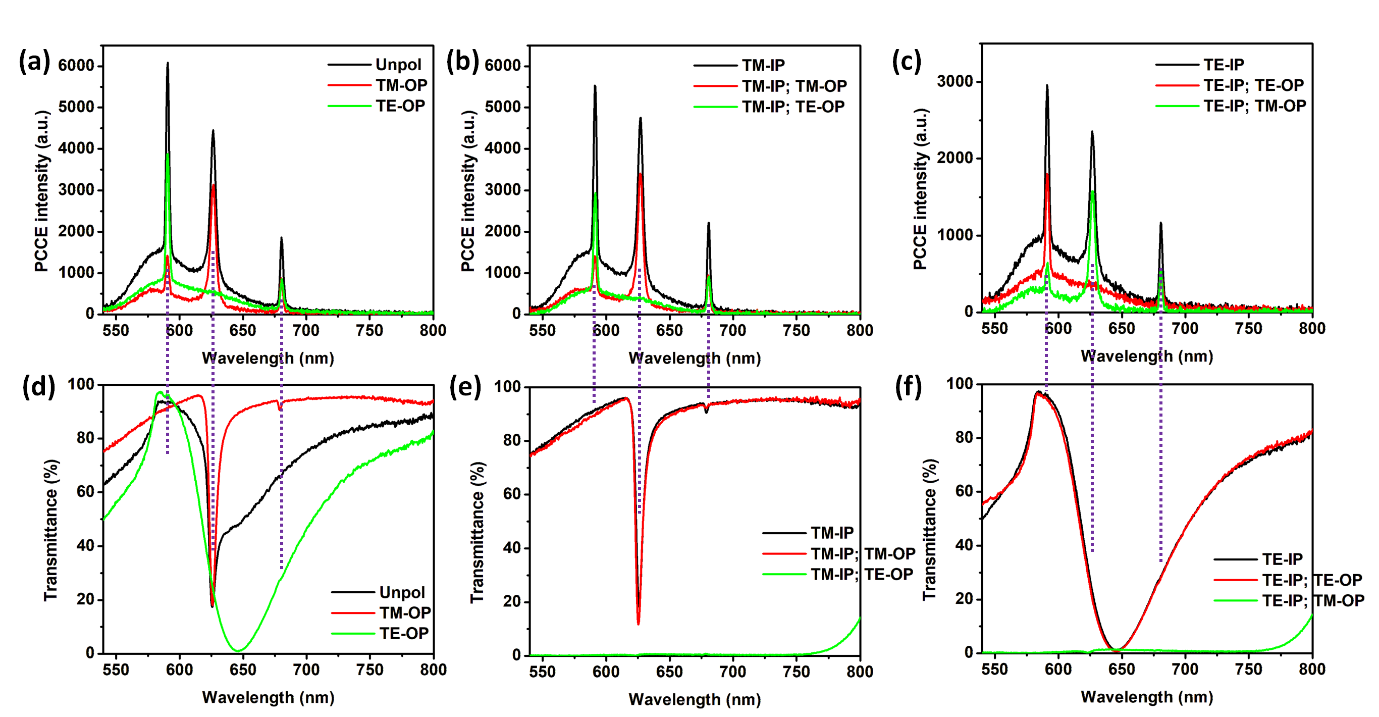


**Figure S6.** **PCCE intensity and transmittance analysis with different polarizers**. (a) PCCE intensity for PC sample at zero degree and changes observed using TM and TE output/emission polarizers. (b) PCCE intensity analysis by inserting TM and TE polarizers at the output, for a fixed input polarizer being TM. (c) PCCE intensity analysis by inserting TM and TE polarizers at the output, for a fixed input polarizer being TE. (d) TE and TM polarized output for unpolarized excitation of the PC with PVA. (e) TE and TM polarized output for TM excitation of the PC with PVA. (f) TE and TM polarized output for TE excitation of the PC with PVA.

The results obtained by inserting excitation and emission polarizers for the sample under consideration is shown in Figure S6b,c. Also, the corresponding transmittance spectral information for different input and output polarizers is shown in Figure S6e,f. In order to enable clear pictorial evaluation, the Figures S6e and S6f are presented vertically below the Figures S6b and S6c respectively. Intriguing observations can be made from the Figures S6b,c,e,f. Firstly, Figure S6b and S6c presents the data for TM and TE input excitation, respectively. It is seen that for TM excitation the modes are largely seen (Figure S6b) as compared to the TE excitation (Figure S6c). This is on account of the intrinsic nature of the radiating dipoles doped in the PVA polymer matrix. In such conformation, the radiating dipoles inherently couple favorably with the TM polarized component of input light (photoselection), in line with earlier understanding of dye coupling in fluorescence spectroscopy. Therefore, the emission intensity is significantly higher for TM excitation vis-à-vis TE excitation.

Further, the radiating dipoles that are excited, emit unpolarized photons at the interface of PC in the micro-nano-environment with wavevectors that are isotropic in nature. These photons selectively couple with the permissible modes of the PC and seep out to the far-field as out-coupled emission. This is understandable by close observations of the occurrence of emission in Figure S6b. From Figure S6b, even though the excitation is TM, the emission is observed and the out-coupled photons couple with both the TE and TM modes of the underlying PC (red) (without an emission polarizer). Nevertheless, introducing an emission polarizer results in selectively filtering the out-coupled photons that couple with only one component of the polarization of light (thereby filtering the other).

Further, very interesting inferences can be drawn by observing the transmittance modes in Figure S6e and the associated fluorescence spectra in Figure S6b. From Figure S6e, we see that for TM excitation, the PC shows a TM resonance at ~630 nm (red). This mode assists in the coupling of emitted photons as seen Figure S6b (red). But we see that for TM excitation, the PC does not show any resonance with TE output polarizer (green line) in Figure S6e [similar observations can be made by careful observation of the modes presented in Figures 6c and 6f]. Hence, these observations validate the understanding of the radiating GMR model. That is, the emitted photons not only move to the far-field by coupling to the modes of the PC, but also excites the GMR of the PC presenting noticeable emission in the far-field. The effective coupling of the photons to the far-field by preserving the polarization attribute of the PCs and the spectral characteristics of the radiating dipole is demonstrated with excellent agreement between the simulated and experimental measurements as detailed in the manuscript.


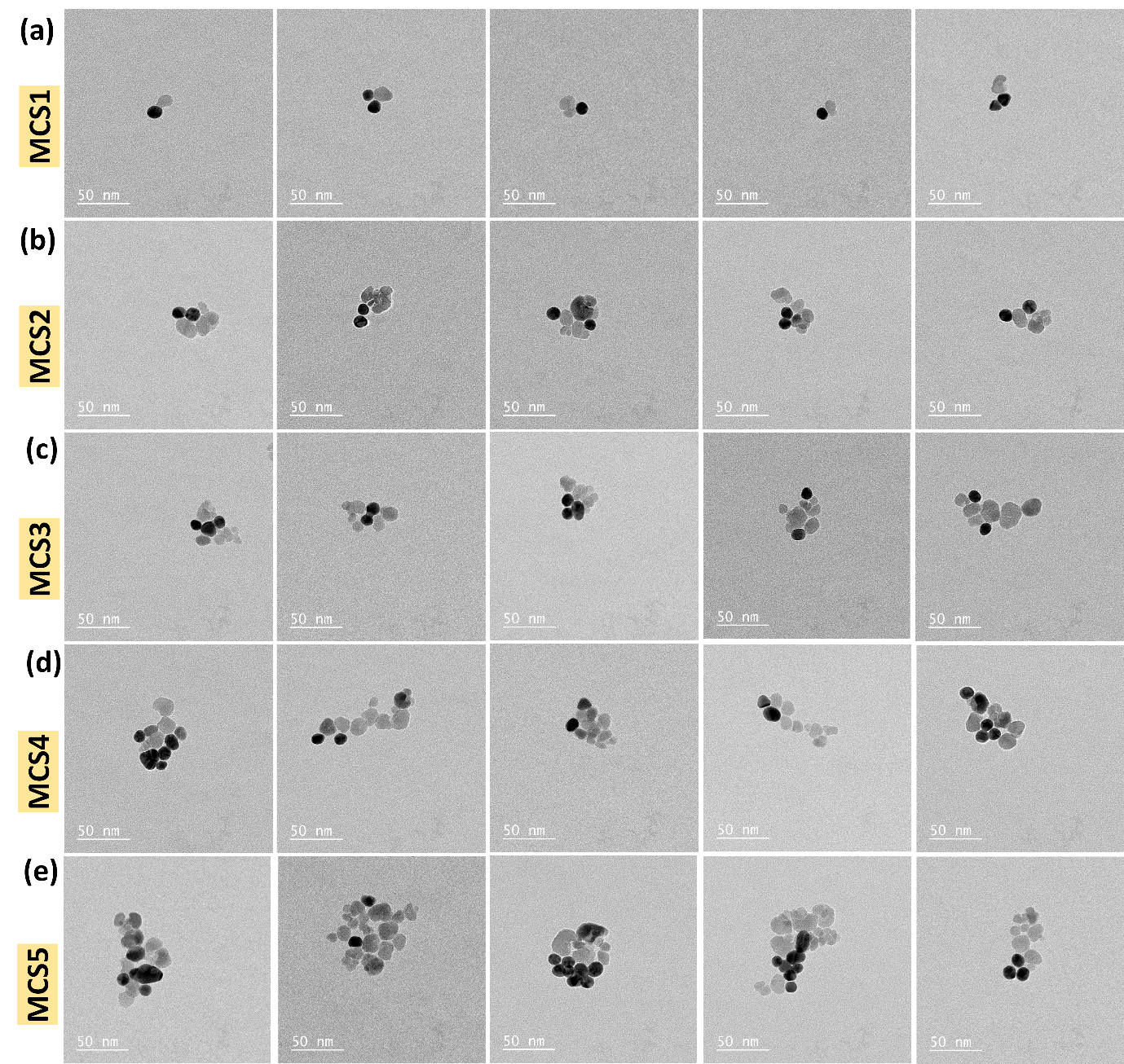


**Figure S7. Transmission electron microscopy analysis of MCSs.** Multiple TEM images for MCSs obtained for different adiabatic cooling timings: (a) MCS1, (b) MCS2, () MCS3, (d) MCS4, (e) MCS5 obtained by subjecting the NPs solution to adiabatic cooling at LN2 temperature (−196 °C) for 15 secs, 30 secs, 1, 2 and 3 min respectively.


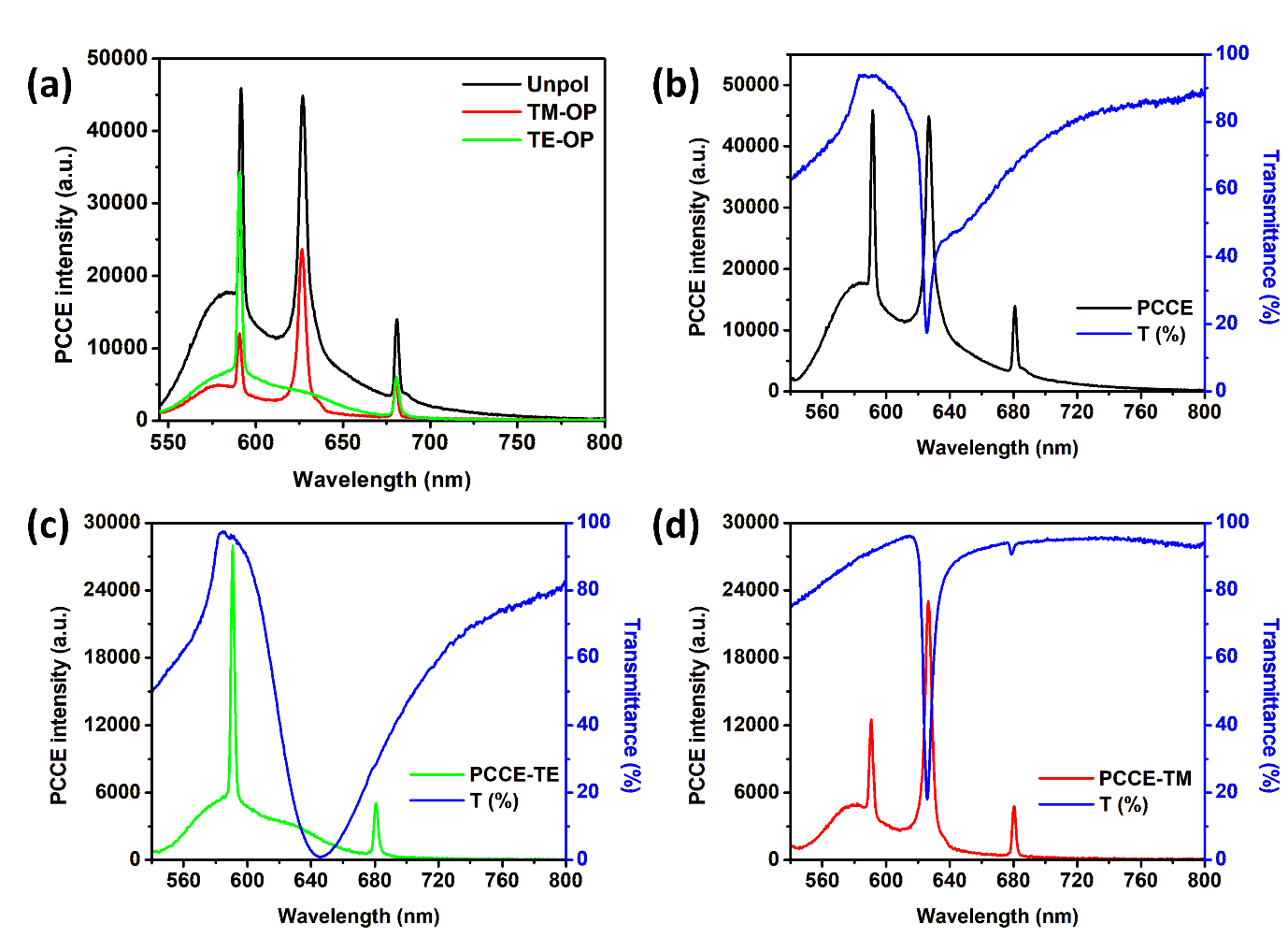


**Figure S8. Analysis of experimental transmittance data with the experimental fluorescence data**. (a) PCCE intensity spectra shown with TE and TM coupled PCCE. Overlap of the experimentally obtained transmittance spectra and the experimentally obtained fluorescence spectra for (b) unpolarized, (b) TM, (c) TE polarized out-coupled spectra. [please note that the Figures S8a is part of figure 4d in the manuscript and is presented here again along with Figure S8b,c,d to present better clarity of the coupling between PC and radiating dipoles].





**Figure S9. Free Space spectra.** The free space fluorescence spectra of RhB recorded experimentally over the glass substrate (under identical conditions as that on PC for effective comparisons) using a 550 nm LWP filter. The different polarizations (TE and TM) of out-coupled emission indicates that the glass substrate coupled fluorescence is not highly polarized.


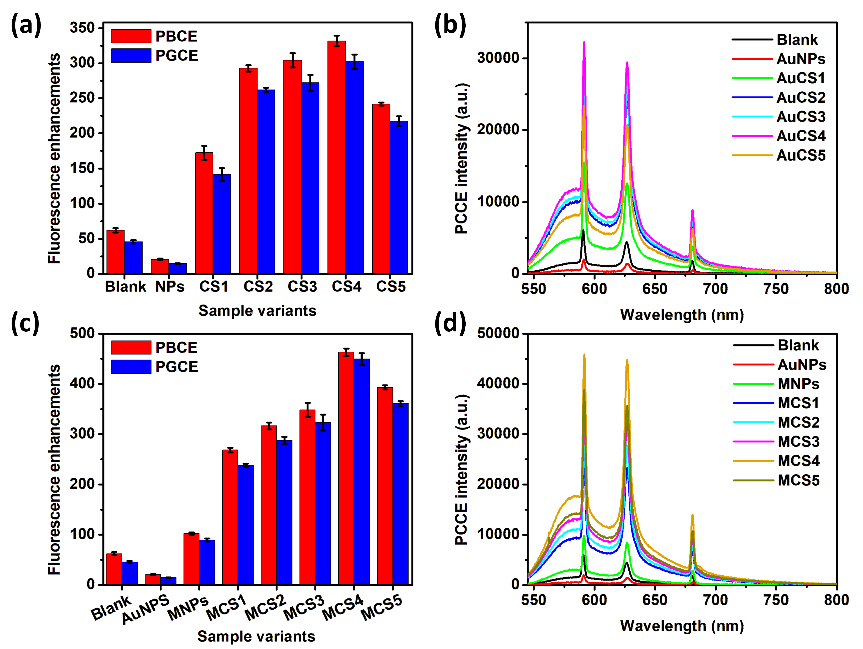


**Figure S10. PCCE enhancements and corresponding emission spectra for all CSs, and MCSs (1-5).** PCCE enhancements are calculated as the ratio of the PCCE intensity counts and the FS intensity counts for (a) all CSs (1-5) variants and (c) MCSs variants (1-5). The respective out-coupled fluorescence spectra is shown in Figure (b) CSs and (d) MCSs respectively. It is to be noted that the emission spectra in Figures S10b and d presents two major peaks around 590 and 630 nm. The former is the photonic-crystal band-edge coupled emission (PBCE) and the latter is the photonic-crystal GMR coupled emission (PGCE). The highest values of these spectra are used to calculate the fluorescence enhancements presented in Figures S10a, c. Also, CSs, refers to plasmonic AuCSs; and MCSs refers to magneto-plasmonic CSs that are made of Au-Fe_3_O_4_ hybrids.


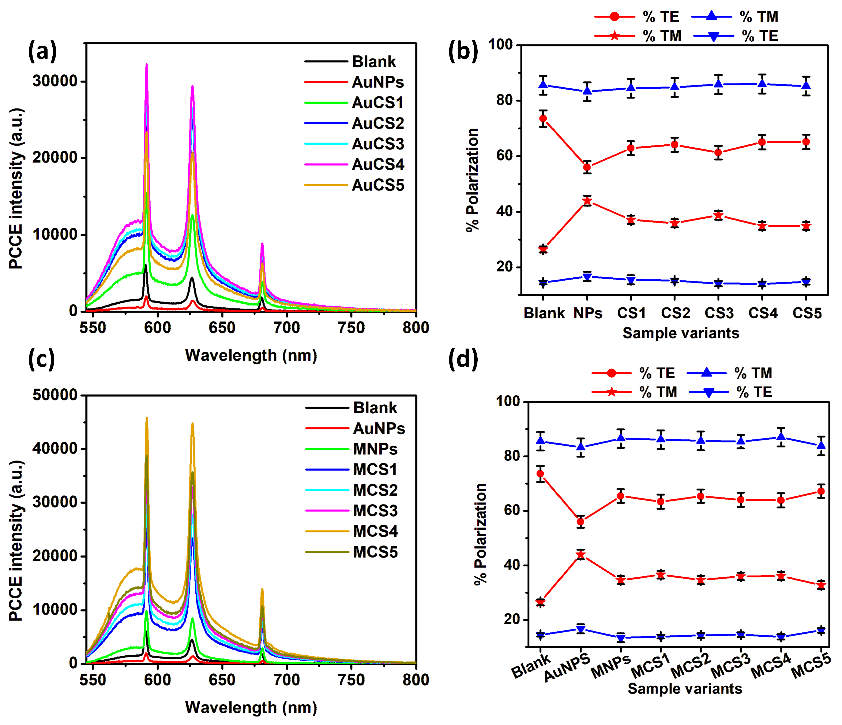


**Figure S11. Polarization property of the out-coupled emission**. The percentage polarization is calculated using the formula %TM = {TM counts ÷ (TM counts + TE counts)}*100. The emission spectra for all the CSs (a) and MCSs (c) are shown along with their respective percentage polarizations on the right side (b, d). The high polarization selectivity of the underlying PC is clearly seen for all the samples under consideration.


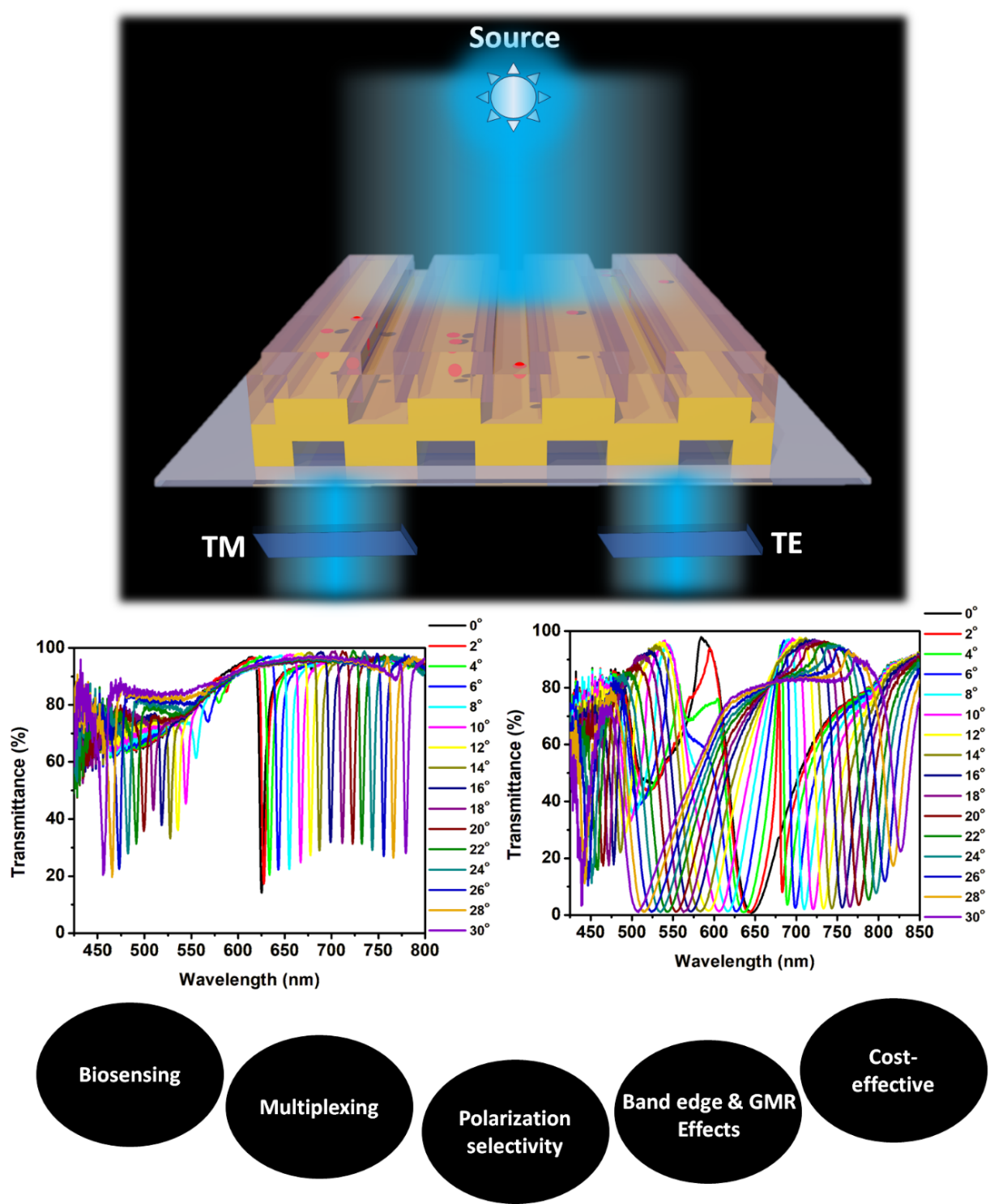


**Figure S12**. **Future scope and perspectives.** The experimental and simulated results presented in this work demonstrates the ability to tune both the TE and TM resonances of the PC to yield high fluorescence enhancements for both the modes. As seen in the above experimental transmittance data, we note that it is possible to explore fluorescent materials emitting at different wavelengths and angles for different polarizations of light (*middle panel*). This enables not only in the multiplexing of the bioassays, but also supports characterization of engineered materials that responds uniquely with a particular polarization of light. Translational applications envisaged with the nano-engineering at the interface of PCs is highlighted in the Figure (*bottom panel*). The appropriate design of nanomaterials with functional architectures and tunable properties would render suitable candidates for detection of disease biomarkers with the development of different types of assays (as the tunable resonances of the PCs would enable multiplexing).
